# A Geospatial Linkage Analysis of Environmental Exposures, Socioeconomic Profile and Health Outcomes Among Women of Reproductive Age in Victoria, Australia

**DOI:** 10.1002/hpja.70241

**Published:** 2026-08-28

**Authors:** Catherine Helenna Mulyadi, Lisa Hui, Heng Jiang, Mari de Leon, J. C. Nacpil, Yichao Wang, Jason Choi, Suzanne Mavoa, Melvin Barrientos Marzan

**Affiliations:** ^1^ College of Computing and Data Science Nanyang Technological University Singapore Singapore; ^2^ Department of Obstetrics, Gynaecology and Newborn Health, Melbourne Medical School University of Melbourne Melbourne Australia; ^3^ Reproductive Epidemiology, Genomic Medicine Theme Murdoch Children's Research Institute Melbourne Australia; ^4^ Department of Obstetrics and Gynaecology Mercy Hospital for Women, Mercy Health Melbourne Australia; ^5^ Department of Obstetrics and Gynaecology The Northern Hospital, Northern Health Melbourne Australia; ^6^ Centre for Alcohol Policy Research La Trobe University Melbourne Australia; ^7^ Melbourne School of Population and Global Health Melbourne Australia; ^8^ Department of Public Health, School of Psychology and Public Health La Trobe University Melbourne Australia; ^9^ Thinking Machines Manila Philippines; ^10^ Prevention Innovation, Population Health Theme Murdoch Children's Research Institute Melbourne Australia; ^11^ Department of Paediatrics, Melbourne Medical School University of Melbourne Melbourne Australia; ^12^ SEED Centre for Lifespan Research Deakin University Geelong Australia; ^13^ Environment Protection Authority Victoria Melbourne Australia

**Keywords:** chronic health burdens, environmental exposures, geospatial linkage, Victoria, women's health

## Abstract

**Issue Addressed:**

Non‐communicable disorders (NCDs) in women of reproductive age (WRA) shape maternal and intergenerational outcomes, yet their area‐level socio‐environmental drivers remain unclear. We examined correlations between socio‐environmental factors and NCDs in WRA.

**Methods:**

We linked 2021 Australian Census sociodemographic and health variables with postcode‐level environmental exposures, including air pollutants (PM_2.5_, NO_2_, CO, SO_2_, formaldehyde and ozone), land surface temperature and greenness. The NCDs (asthma, diabetes, cancer, mental health and cardio‐renal disease) were expressed per 1000 WRA. Spatial clustering (Moran's *I*) and socioeconomic gradients (Wagstaff Concentration Index) were assessed, alongside geospatial regression and clustering analyses.

**Results:**

NCDs among WRA showed strong socioeconomic patterning. Most disadvantaged postcodes recorded the highest composite NCD burden (323.0 and 227.3 per 1000 in the most disadvantaged and advantaged postcodes respectively), with the steepest inequality for diabetes (Wagstaff = −0.16). Environmental exposures demonstrated spatial clustering but weak associations with health outcomes. Notably, peri‐urban and fringe postcodes experienced higher heat exposure coinciding with socioeconomic disadvantage. Inner Melbourne (with lower heat exposure and more socioeconomic advantage) showed lower NCD burden despite elevated NO_2_ and PM_2.5_.

**Conclusion:**

NCDs among WRA in Victoria are strongly patterned by socioeconomic disadvantage with geographic clustering by environmental exposures. These inequities persist even where most air quality measures remain within regulatory limits, underscoring the disproportionate burden faced by disadvantaged areas.

**So What:**

Health promotion should prioritise disadvantaged peri‐urban and regional communities through proactive, place‐based policies that address both environmental conditions and the social systems that influence how and for whom those conditions become harmful.

## 1 Background

Women of reproductive age (WRA, 15–49 years) represent a critical population in public health, recognised as a priority for prevention and equity in policies such as the National Women's Health Strategy 2020–2030. This national framework highlights the reproductive years as a critical window for addressing non‐communicable disease (NCD) risk and social determinants [1]. Similarly, Victoria's Women's Sexual and Reproductive Health Plan 2022–2030 [2] emphasises reducing inequities and improving access to services, particularly for disadvantaged and regional communities.

The burden of NCDs among WRA outside pregnancy remains understudied [3]. This gap is concerning given the rising prevalence of NCDs and their implications for both women's long‐term health and intergenerational outcomes. For example, the incidence of gynaecological conditions has increased among younger WRA, particularly among women aged 20–24 years, with much of this rise driven by polycystic ovary syndrome (PCOS), a condition associated with elevated lifelong risks of cardiovascular disease, diabetes and infertility [4]. At the same time, environmental exposures are increasingly recognised as important determinants of health in this population. Exposure to air pollution and extreme heat has been linked to cardiometabolic strain, while limited access to green space has been associated with poorer mental health and psychological distress [5, 6, 7]. These environmental conditions are also relevant to infants and young children, who are particularly vulnerable because of their limited thermoregulatory capacity, developing respiratory systems and reduced ability to behaviourally respond to heat and pollution exposure [7]. As women of reproductive age often live in shared environments with infants and young children, especially during pregnancy and early parenting years, inequities in environmental conditions may have implications across multiple stages of the life course. Yet, studies examining how environmental exposures intersect with social disadvantage among WRA remain scarce.

Women's health research has traditionally focused on maternal or perinatal outcomes, often within capital cities and major urban centres. However, the rising burden of chronic conditions among WRA [4] signals the importance of expanding the women's health research scope to understand how environmental exposures contribute to health outcomes including women in peri‐urban and regional areas, whose experiences were not captured by the existing urban‐centric research focus. In Australia, where climate variability and urban heat intensification are increasingly salient threats [8], the spatial distribution of environmental exposures and their association with chronic disease remains poorly mapped, particularly among WRA.

Spatial epidemiological methods enable identification of spatial clusters and socioeconomic gradients by merging health and environmental data at local scales such as neighbourhoods, zip codes, or counties [9, 10]. Applying these methods to WRA can provide new opportunities to reduce the health burden by identifying geographic hotspots of disease and their underlying drivers. Yet to date, few studies have linked area‐level environmental data to health outcomes in a way that captures both spatial structure and social stratification for WRA. These priorities highlight the need for evidence on how disadvantage and environmental conditions shape the health of WRA beyond pregnancy.

Our study aimed to characterise the spatial and socioeconomic patterns of environmental exposures and NCD health outcomes among WRA in Victoria, Australia. Using a geospatial data linkage approach, we examined how postcode‐level environmental conditions, including air pollution, heat and vegetation, correlate with the prevalence of long‐term health burden across Socio‐Economic Indexes for Areas (SEIFA)‐defined socioeconomic strata and whether these patterns demonstrate spatial clustering or systematic inequality.

## 2 Methods

This study is a geospatial ecological analysis examining the distribution of long‐term health outcomes/NCDs among WRA, along with sociodemographic and environmental characteristics, across postcodes in Victoria, Australia. We followed the Spatial Life course Epidemiology Reporting Standards (ISLE‐ReSt) [11] (Table S1). We obtained ethical approval for this study from the Human Research Ethics Committee of Mercy Health (Ref. no 2023‐044). Postal Areas (POAs) were used as the unit of analysis. POAs are Australian Bureau of Statistics (ABS)‐defined approximations of Australia Post postcodes, constructed by allocating whole Mesh Blocks, the smallest ABS geographic unit, typically containing 30–60 dwellings, to a postcode [12]. As a result, metropolitan postcodes' total land areas are generally smaller than rural postcodes. This structure provides a more stable population denominator, supports comparability across areas and reduces small‐area instability in health rates.

### 2.1 Data Sources

#### 2.1.1 Health, Sociodemographic and Economic Data

Demographic, socioeconomic and health‐related variables were obtained from the 2021 Australian Census and aggregated at the postcode (POA) level [13]. WRA were defined as females aged 15–49 years [14]. Variables included total population counts, the proportion of WRA, proportion of Aboriginal and Torres Strait Islander peoples, country of birth, language spoken at home and citizenship status [15]. Aboriginal and Torres Strait Islander population measures came from the Australian Bureau of Statistics (ABS) Census and served as aggregated postcode‐level indicators only. We used these data in secondary analysis of publicly available aggregate Census data and did not access identifiable individual‐level First Nations data or Indigenous‐specific datasets. We also extracted indicators of long‐term health conditions/NCDs from the 2021 Australian Census, which are diagnosed by clinicians and last 6 months or longer, based on self‐reported diagnoses among WRA, including asthma and other respiratory conditions, cancer, diabetes, mental health conditions and cardiovascular, renal or cerebrovascular diseases (including stroke), expressed per 1000 WRA and combined into a composite burden score [15, 16]. Socioeconomic variables included median age, housing and income‐related measures, household composition and the Index of Relative Socio‐economic Advantage and Disadvantage (SEIFA‐IRSAD), where lower values reflect greater disadvantage [13].

#### 2.1.2 Environmental Data

We sourced environmental exposure data from multiple platforms and models, harmonised to the postcode level across Victoria. Land surface temperature (maximum and median) and normalised difference vegetation index (NDVI) were derived from Landsat satellite imagery accessed via Google Earth Engine (GEE). Fine particulate matter (PM_2.5_) was obtained from the CSIRO Chemical Transport Model, providing surface‐level estimates in μg/m^3^. Nitrogen dioxide (NO_2_) concentrations were based on the land‐use regression model [17], incorporating satellite‐derived tropospheric NO_2_ and ground‐based calibration to estimate surface‐level exposure. We extracted atmospheric columnar gas concentrations for carbon monoxide (CO), sulphur dioxide (SO_2_), formaldehyde (HCHO) and ozone (O_3_) from the Sentinel‐5 Precursor (Sentinel‐5P) satellite, which carries the TROPOspheric Monitoring Instrument (TROPOMI) [18]. These were expressed as mol/m^2^ for trace gases and Dobson Units for ozone, representing total atmospheric column content rather than surface exposure. Further details on our methodologies are included in the Supporting Information.

To smooth inter‐annual variability, we averaged all environmental indicators across 4 years: 2019, 2020, 2021 and 2022. This multi‐year average was used in all analyses to reflect typical exposure conditions during the pre‐pandemic and pandemic period.

### 2.2 Statistical Analysis

We mapped postcode‐level environmental exposures (e.g., air pollutants, greenness, land surface temperature) and health vulnerability indicators (e.g., composite burden of long‐term conditions among women of reproductive age) across Victoria. To assess spatial clustering, we calculated Global Moran's *I* for 18 environmental and health‐related variables using a queen contiguity spatial weights matrix with a 0.0001 snapping threshold. We reported Moran's *I* values, *z*‐scores and *p*‐values to detect spatial autocorrelation. This step determines whether the data exhibited spatial autocorrelation, which require the use of geospatial regression models to account for geographic structure and avoid biased estimates.

Next, we used geospatial regression to model the categorised NCDs and the composite long‐term condition burden. We included environmental and social indicators a priori, drawing on subject matter expertise and evidence of links to chronic disease [5, 6, 7]. Our predictors included:

- *Population characteristics*: Proportion of First Nations, born in Australia, born elsewhere, English‐only speakers, other language speakers and Australian citizens.
- *Socioeconomic indicators*: Index of Relative Socio‐economic Advantage and Disadvantage (IRSAD).
- *Housing and income*: Median age, mortgage repayment, rent and household income
- *Environmental exposures*: Normalised difference vegetation index (NDVI), NO_2_, atmospheric water vapour, formaldehyde, ozone, PM_2.5_ and land surface temperature (maximum and median).

To assess socioeconomic inequalities in exposure and burden, we computed the Wagstaff Concentration Index (C) using ranked SEIFA IRSAD scores. A negative C indicates that the burden is concentrated in more disadvantaged areas. Finally, we applied k‐means clustering due to its computational efficiency and scalability [19] to group postcodes based on 17 environmental and sociodemographic variables. We ran separate models for Metro Melbourne and Regional Victoria, each with four clusters. We summarised cluster characteristics and tested group differences using Kruskal‐Wallis and Dunn's post hoc tests with Bonferroni correction. All geospatial and statistical analysis was performed in R (R version 4.0.1, http://www.r‐project.org).

As a sensitivity analysis, we refitted the spatial lag models separately within urbanicity strata, contrasting Metropolitan Melbourne with Regional Victoria and, in a secondary analysis, an inner‐city, suburban and regional three‐tier split, rather than adjusting for metropolitan status within a single pooled model. Each stratified model used the same variables and specification as the main analysis (further details in the Supporting Information).

## 3 Results

### 3.1 Spatial Patterning of Health Conditions

**FIGURE 1:**
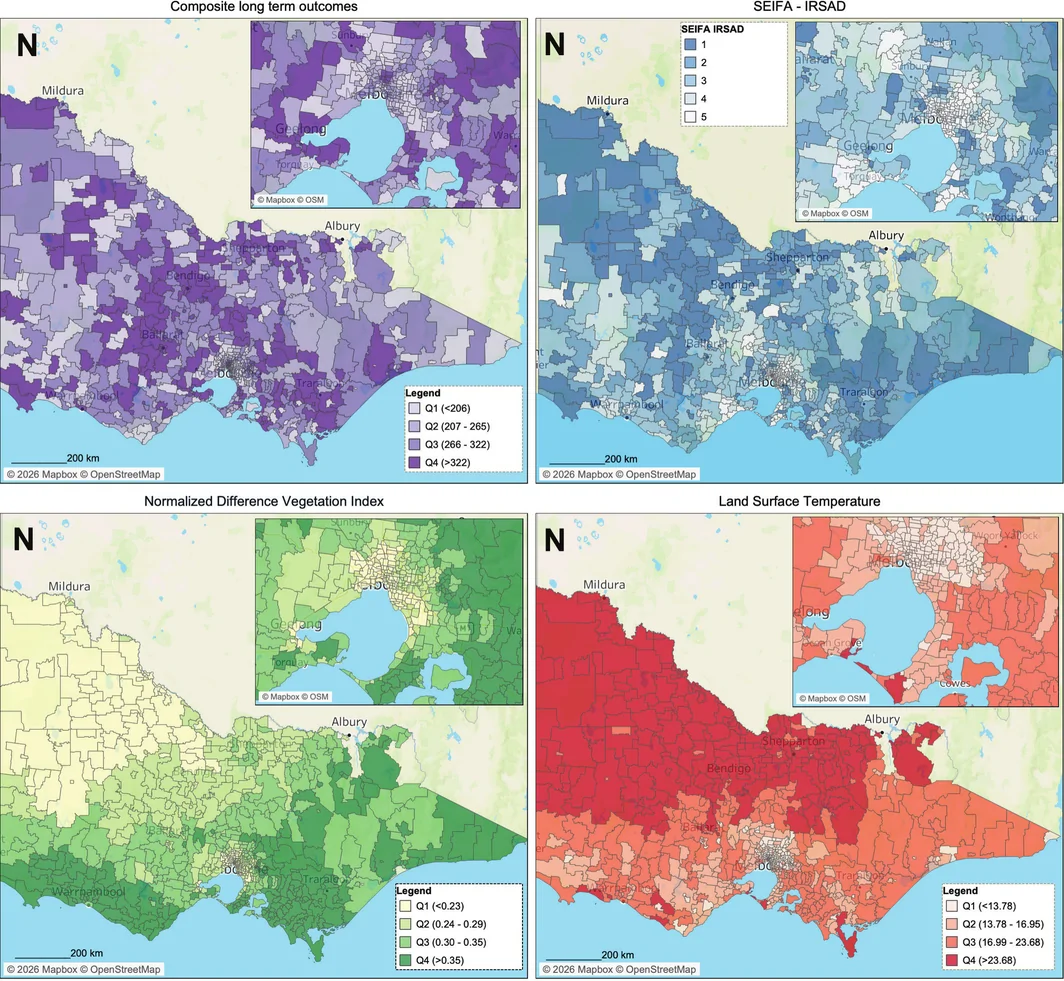
Prevalence of self‐reported non‐communicable diseases among WRA per 1000 population, socioeconomic status, greenness and land surface temperature by postcode. *Inlet map is metropolitan Melbourne. Q, Quintile; SEIFA‐IRSAD, Socio‐Economic Indexes for Areas—Index of Relative Socio‐economic Advantage and Disadvantage.

Composite long‐term health outcomes among WRA showed substantial spatial variation across Victoria, closely aligned with socioeconomic deprivation, greenness and land surface temperature (LST) (Figure 1). The highest NCD burden was in the north‐west and outer‐regional areas, which consistently corresponded with the highest level of socioeconomic deprivation (Quintile 1). Notably, a high NCD burden was also evident in parts of central Victoria, a region that generally exhibited greater socioeconomic advantage (Quantile 5), suggesting the influence of factors beyond broad socioeconomic indicators in this area. In contrast, inner‐Melbourne and coastal regions generally showed a lower burden alongside higher socioeconomic status. Environmental characteristics also varied geographically. Greenness was lowest in metropolitan Melbourne and peri‐urban corridors, and more prominent in eastern and south‐western rural areas. LST was higher in inland and north‐western Victoria, and lower around Melbourne and vegetated zones.

### 3.2 Spatial Patterning of Environmental Exposures

**FIGURE 2:**
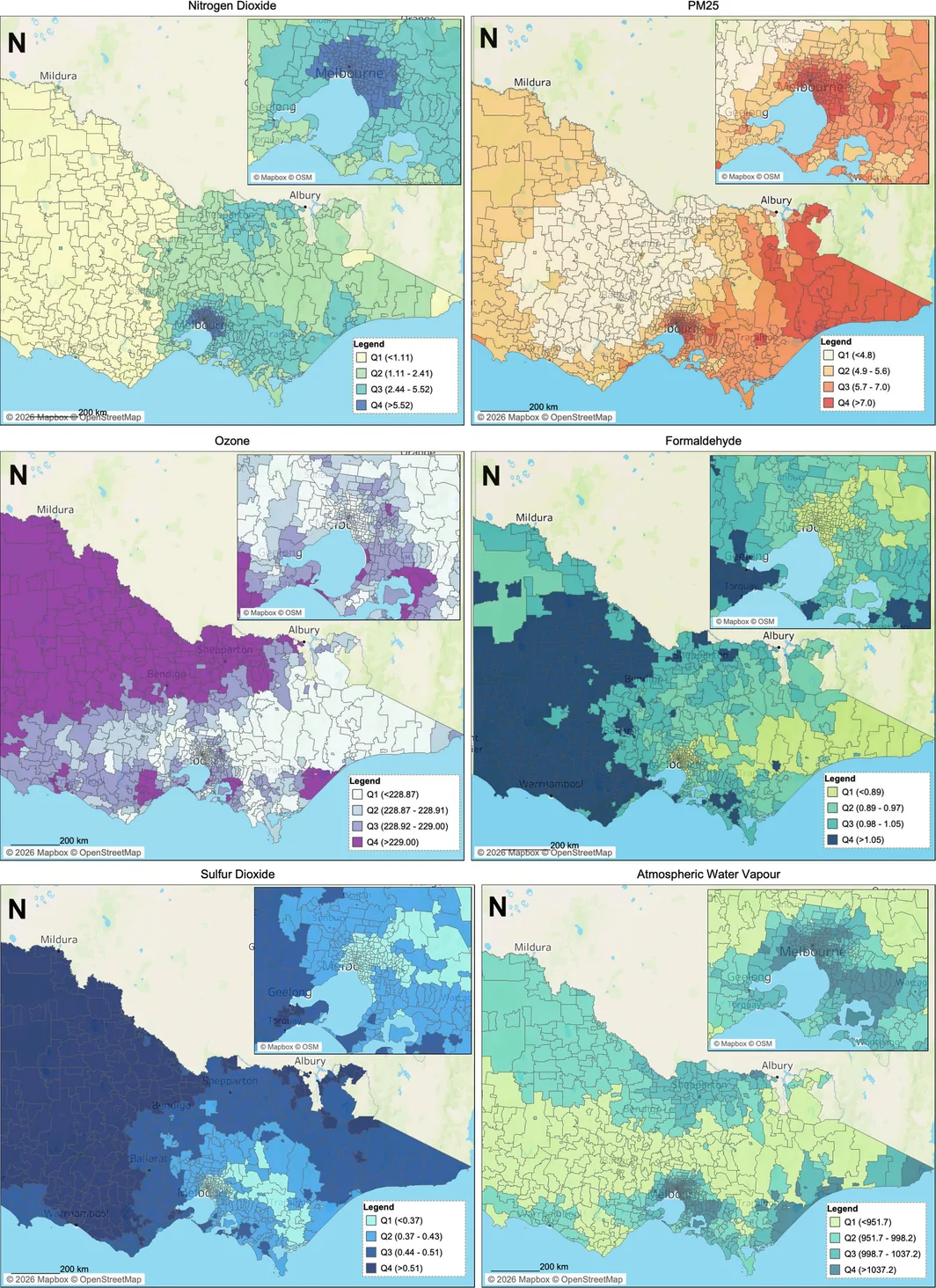
Geographic variation of air pollutants exposure across Victoria. *Inlet map is metropolitan Melbourne. Q, Quintile.

Clustered air quality patterns were observed in the environmental exposures' distribution visualisations summarised in Figure 2. Nitrogen dioxide (NO_2_) and PM_2.5_ were higher in Greater Melbourne, especially in densely populated and industrial areas. Formaldehyde and sulphur dioxide (SO_2_) were elevated in parts of outer‐metropolitan and regional Victoria, while ozone levels were higher in western and north‐western postcodes. Atmospheric water vapour was more concentrated in coastal and south‐eastern areas.

**FIGURE 3:**
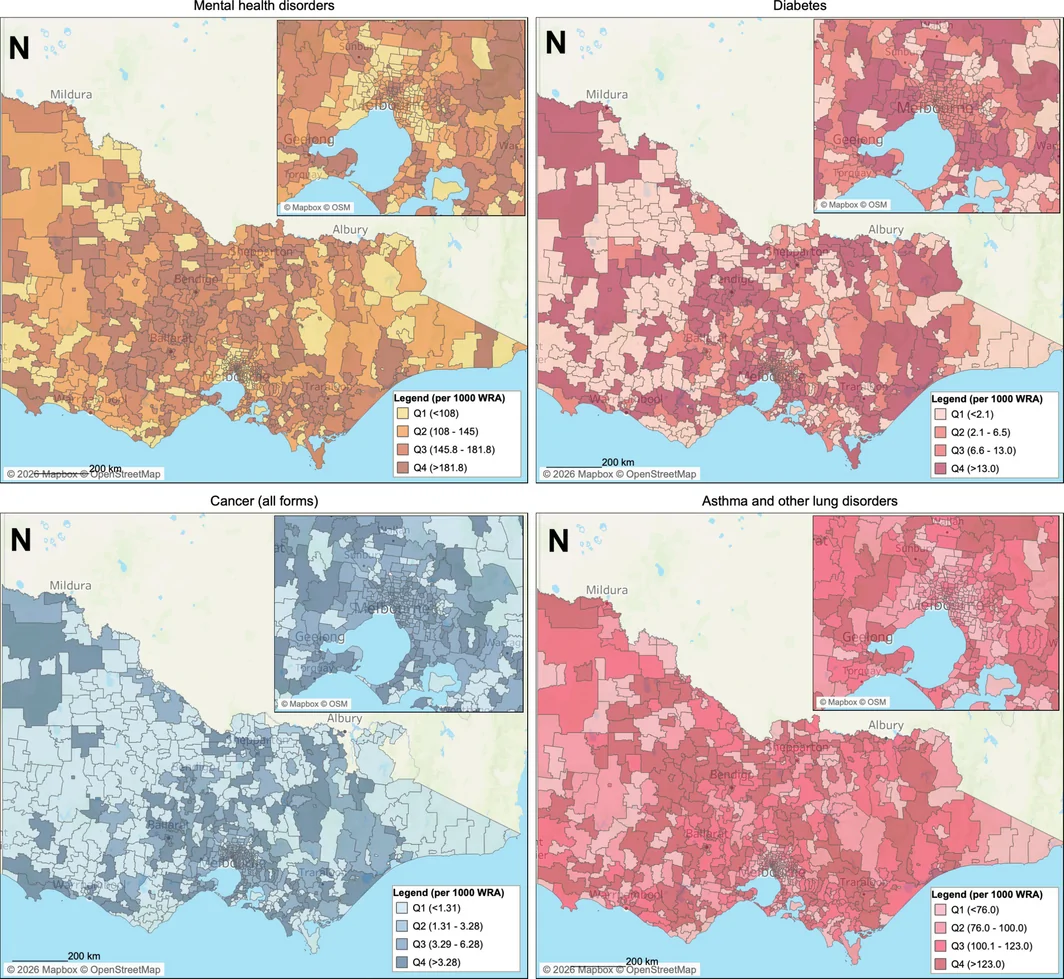
Prevalence of self‐reported non‐communicable diseases among WRA per 1000 population by post‐code. *Inlet map is metropolitan Melbourne. Q, Quintile.

The maps reveal substantial geographic variation in the burden of NCDs across Victoria (Figure 3). Cancer and diabetes prevalences appear highly concentrated in both metropolitan Melbourne and selected rural regions, while mental health disorders and respiratory conditions such as asthma show a more diffused pattern across regional and outer suburban areas.

### 3.3 Socio‐Economic Gradient and Geospatial Associations

Table 1 presents the summary statistics, socioeconomic gradients and spatial patterns for NCDs among WRA, as well as environmental exposures across Victorian postcodes. A consistent socioeconomic gradient was observed across most health outcomes. The burden of composite long‐term conditions was highest in the most disadvantaged areas (SEIFA Quintile 1: 323.0 per 1000 WRA) and declined steadily across quintiles, reaching 227.3 in the most advantaged areas (Quintile 5). Similar patterns were seen for mental health (176.9 vs. 127.9), asthma and other lung conditions (125.4 vs. 85.1) and diabetes (11.8 vs. 6.1). The Wagstaff Concentration Index confirmed a disadvantage‐inclined distribution for these outcomes with the steepest inequality for diabetes (−0.16), followed by cardiovascular/renal disease (−0.08), composite outcomes (−0.07) and mental health conditions (−0.06). Cancer showed no clear pattern by socioeconomic status (Wagstaff = +0.06, *p* = 0.28).

**TABLE 1:** Prevalence of long‐term health conditions among women of reproductive age (per 1000 WRA) and environmental exposures across Victorian postcodes, by Index of Relative Socio‐economic Advantage and Disadvantage (IRSAD) quintile, with Wagstaff Concentration Indices and Global Moran's *I*: Victoria, Australia, 2021.

	IRSAD 1 (most disadvantaged)	IRSAD 2	IRSAD 3	IRSAD 4	IRSAD 5 (most advantaged)	*p*	Wagstaff concentration index	Moran's *I*
Long‐term health conditions (per 1000 WRA)								
Composite long‐term conditions	323.02	260.31	245.40	238.44	227.27	0.0000	**−0.0648**	0.10*
Mental health conditions	176.93	149.15	134.93	133.25	127.87	0.0000	**−0.0627**	0.10*
Cardiovascular, renal or cerebrovascular conditions (including stroke)	5.42	2.95	3.97	3.57	3.26	0.0078	**−0.0736**	0.08*
Asthma and other lung conditions	125.36	92.82	94.91	90.86	85.12	0.0000	**−0.0647**	0.07*
Cancer	3.55	3.82	4.12	3.99	4.91	0.2779	0.0548	0.03
Diabetes	11.76	11.53	7.46	6.78	6.11	0.0004	**−0.1565**	0.02
Environmental exposures								
Greenness, Normalised Difference Vegetation Index (NDVI), unitless	0.28	0.30	0.31	0.31	0.26	0.0000	0.0024	0.88*
Nitrogen dioxide (NO_2_), ppb	2.47	2.23	2.72	4.05	7.24	0.0000	0.2398	0.97*
Carbon monoxide (CO), μmol/m^2^/s	0.0209	0.0208	0.0207	0.0208	0.0211	0.0000	0.0012	0.82*
Atmospheric water vapour, Dobson Units	983.78	970.70	971.64	981.77	1026.19	0.0000	0.0077	0.80*
Formaldehyde (HCHO), mol/m^2^	1.00	1.00	1.00	0.97	0.89	0.0000	−0.0209	0.90*
Ozone (O_3_), Dobson Units	228.98	228.97	228.93	228.92	228.89	0.0000	−0.0001	0.86*
Sulphur dioxide (SO_2_), Dobson Units	0.48	0.47	0.46	0.43	0.38	0.0000	−0.0434	0.95*
Fine particulate matter (PM_2.5_), μg/m^3^	5.82	5.79	5.69	6.05	7.26	0.0000	0.0394	0.93*
Land surface temperature (LST), maximum, °C	22.44	22.59	20.05	17.07	14.01	0.0000	−0.0902	0.85*
Land surface temperature (LST), median, °C	12.53	11.59	10.48	8.83	8.04	0.0000	−0.0902	0.92*

*Note: Unit of analysis*: Australian Bureau of Statistics (ABS) Postal Areas (POAs), approximating postcodes, in Victoria, Australia. Cell values are the mean of the postcode‐level measure within each IRSAD quintile. Health outcomes are self‐reported, clinician‐diagnosed long‐term conditions (lasting 6 months or longer) from the 2021 Census, expressed per 1000 WRA. The composite measure is the sum of the five condition groups, so a woman with more than one condition contributes to more than one group. IRSAD quintiles: quintile 1 = most disadvantaged, quintile 5 = most advantaged postcodes. *p*‐value: test of the null hypothesis of no socioeconomic gradient across IRSAD quintiles. Wagstaff Concentration Index: ranges from −1 to +1. Negative values indicate the outcome or exposure is concentrated in more disadvantaged postcodes; positive values indicate concentration in more advantaged postcodes; 0 indicates no socioeconomic gradient. *Moran's I*: Global Moran's *I* computed with a queen contiguity spatial weights matrix (0.0001 snapping threshold). Values near 0 indicate no spatial autocorrelation and values near 1 indicate strong spatial clustering. *Exposure sources*: NDVI and LST from Landsat imagery (Google Earth Engine), averaged 2018–2021; PM_2.5_ from the CSIRO Chemical Transport Model (surface‐level); NO_2_ from a national satellite‐based land‐use regression model (surface‐level); CO, HCHO, O_3_, SO_2_ and atmospheric water vapour from Sentinel‐5P TROPOMI and representing total atmospheric column content rather than surface‐level exposure.

Abbreviations: ABS, Australian Bureau of Statistics; CO, carbon monoxide; HCHO, formaldehyde; IRSAD, Index of Relative Socio‐economic Advantage and Disadvantage (a SEIFA index; higher scores indicate greater advantage); LST, land surface temperature; NDVI, Normalised Difference Vegetation Index (range −1 to +1; higher values indicate greater greenness); NO_2_, nitrogen dioxide; O_3_, ozone; PM_2.5_, fine particulate matter with an aerodynamic diameter < 2.5 μm; POA, Postal Area; ppb, parts per billion; SEIFA, Socio‐Economic Indexes for Areas; SO_2_, sulphur dioxide; TROPOMI, TROPOspheric Monitoring Instrument; WRA, women of reproductive age (females aged 15–49 years).

* *p* < 0.05.

Environmental exposures varied by SEIFA quintile but did not always follow a clear gradient. Greenness was highest in the middle quintiles and lower at both extremes, suggesting a non‐linear relationship with area‐level socioeconomic status. LST, both maximum and median, showed an inverse relationship with advantage, with disadvantaged areas experiencing higher heat exposure (Wagstaff = −0.09). Air quality indicators diverged in their patterns: NO_2_ and PM_2.5_ were highest in more advantaged areas (Wagstaff = +0.24 and +0.04, respectively). Formaldehyde and SO_2_ showed weak gradients favouring higher exposure in lower SEIFA quintiles, though these differences were small. Spatial autocorrelation was strongest for NO_2_ (Moran's *I* = 0.97), PM_2.5_ (0.93) and NDVI (0.88), indicating strong spatial clustering of these exposures. Among health outcomes, composite and mental health conditions had mild but significant spatial dependence (Moran's *I* = 0.10), supporting the notion of geographic patterning in health burden across Victorian postcodes.

Table 2 presents the results of spatially adjusted geospatial regression models assessing associations between sociodemographic composition, socioeconomic status and environmental exposures with long‐term health conditions among women of reproductive age across Victorian postcodes. Postcodes with higher proportions of Australian‐born residents, English‐only speakers and Australian citizens showed consistently higher rates of asthma, mental health conditions and composite long‐term burden, a pattern clearly demonstrated by the significant association with Australian citizenship (*β* = 4.18; 95% CI: 3.12, 5.23). In contrast, higher proportions of people born overseas or speaking a language other than English were associated with lower burdens of mental health (*β* = −1.96; 95% CI: −2.47, −1.45) and composite outcomes (*β* = −3.20; 95% CI: −3.89, −2.51). The higher proportion of First Nations residents was positively associated with increased mental health burden (*β* = 5.10; 95% CI: 0.27, 9.93). Higher median age was associated with lower prevalence of several outcomes, including asthma (*β* = −0.78; 95% CI: −1.35, −0.21) and diabetes (*β* = −0.23; 95% CI: −0.39, −0.07). Conversely, areas with higher median rent showed higher rates of all health conditions, with the strongest association observed for mental health conditions (*β* = 0.16; 95% CI: 0.08, 0.25). Greater socioeconomic advantage, as measured by IRSAD, was inversely associated with nearly all outcomes. The strongest association was seen for composite burden (*β* = −12.10; 95% CI: −15.26, −8.93), followed by mental health and asthma.

**TABLE 2:** Spatial lag regression coefficients (*β*) and 95% confidence intervals for associations of sociodemographic composition, socioeconomic status and environmental exposures with long‐term health conditions among women of reproductive age, per 1000 WRA: Victorian postcodes, Australia, 2021.

Predictor	Asthma and other lung conditions	Cancer	Diabetes	Cardiovascular, renal or cerebrovascular conditions (including stroke)	Mental health conditions	Composite long‐term conditions
Sociodemographic composition (proportion of the postcode population, %)						
Aboriginal and Torres Strait Islander (First Nations) peoples	1.87 (−1.31, 5.06)	−0.07 (−0.42, 0.27)	−0.47 (−1.38, 0.44)	−0.15 (−0.53, 0.23)	5.10 (0.27, 9.93)*	6.29 (−0.36, 12.94)
Born in Australia	1.51 (1.13, 1.89)***	0.05 (0.01, 0.09)*	−0.03 (−0.14, 0.08)	0.03 (−0.02, 0.07)	2.08 (1.50, 2.65)***	3.62 (2.83, 4.40)***
Born overseas	−1.20 (−1.62, −0.78)***	−0.02 (−0.07, 0.02)	0.06 (−0.06, 0.18)	−0.00 (−0.05, 0.05)	−1.60 (−2.24, −0.96)***	−2.73 (−3.61, −1.85)***
Speaks English only at home	1.36 (1.06, 1.67)***	0.04 (0.01, 0.08)*	−0.01 (−0.10, 0.08)	0.03 (−0.01, 0.07)	2.11 (1.65, 2.58)***	3.56 (2.93, 4.19)***
Speaks a language other than English at home	−1.21 (−1.54, −0.88)***	−0.03 (−0.07, 0.00)	0.03 (−0.06, 0.13)	−0.02 (−0.06, 0.02)	−1.96 (−2.47, −1.45)***	−3.20 (−3.89, −2.51)***
Australian citizen	1.90 (1.39, 2.40)***	0.09 (0.03, 0.14)**	−0.02 (−0.17, 0.13)	0.06 (−0.00, 0.12)	2.18 (1.41, 2.96)***	4.18 (3.12, 5.23)***
Selected postcode medians						
Median age of persons, years	−0.78 (−1.35, −0.21)**	−0.14 (−0.20, −0.08)***	−0.23 (−0.39, −0.07)**	−0.17 (−0.24, −0.11)***	−0.04 (−0.90, 0.83)	−1.27 (−2.46, −0.09)*
Median monthly mortgage repayment, AUD	0.01 (0.00, 0.02)*	0.00 (0.00, 0.00)***	0.00 (−0.00, 0.01)	0.00 (0.00, 0.00)***	0.01 (−0.00, 0.03)	0.03 (0.01, 0.06)**
Median weekly rent, AUD	0.10 (0.05, 0.16)***	0.02 (0.02, 0.03)***	0.02 (0.01, 0.04)**	0.02 (0.01, 0.02)***	0.16 (0.08, 0.25)***	0.32 (0.21, 0.44)***
Median weekly total household income, AUD	0.02 (0.01, 0.03)**	0.00 (−0.00, 0.00)	0.00 (−0.00, 0.01)	0.00 (−0.00, 0.00)	0.02 (−0.00, 0.04)	0.04 (0.01, 0.07)**
Socioeconomic status (SEIFA)						
IRSAD score (higher = more advantaged)	−4.68 (−6.18, −3.18)***	−0.06 (−0.22, 0.10)	−1.19 (−1.62, −0.77)***	−0.50 (−0.68, −0.32)***	−5.77 (−8.04, −3.51)***	−12.10 (−15.26, −8.93)***
Environmental exposures						
Greenness, NDVI (unitless)	47.41 (0.82, 94.00)*	7.68 (2.64, 12.71)**	−1.15 (−14.46, 12.16)	2.68 (−2.91, 8.27)	21.59 (−49.13, 92.31)	77.01 (−20.47, 174.49)
Nitrogen dioxide (NO_2_), ppb	−0.33 (−2.27, 1.61)	0.17 (−0.04, 0.38)	−0.14 (−0.70, 0.41)	0.24 (0.01, 0.47)*	2.39 (−0.56, 5.33)	2.43 (−1.63, 6.48)
Atmospheric water vapour, Dobson Units	−0.01 (−0.07, 0.05)	−0.00 (−0.01, 0.00)	−0.00 (−0.02, 0.01)	0.00 (−0.00, 0.01)	−0.08 (−0.18, 0.01)	−0.10 (−0.23, 0.03)
Formaldehyde (HCHO), mol/m^2^	20.54 (−35.46, 76.53)	−8.27 (−14.33, −2.22)**	−1.07 (−17.06, 14.91)	−7.54 (−14.28, −0.79)*	−5.72 (−90.68, 79.25)	−5.04 (−121.52, 111.44)
Ozone (O_3_), Dobson Units	−12.24 (−45.76, 21.29)	−5.54 (−9.18, −1.91)**	−0.09 (−9.68, 9.49)	−2.81 (−6.84, 1.22)	−2.54 (−53.49, 48.41)	−23.48 (−93.46, 46.49)
Fine particulate matter (PM_2.5_), μg/m^3^	−2.37 (−4.79, 0.06)	0.06 (−0.20, 0.32)	−0.11 (−0.80, 0.58)	0.18 (−0.11, 0.47)	−4.37 (−8.06, −0.68)*	−6.00 (−10.97, −1.03)*
Land surface temperature (LST), maximum, °C	−0.47 (−1.13, 0.19)	−0.10 (−0.17, −0.03)**	0.19 (0.00, 0.38)*	−0.07 (−0.15, 0.01)	−0.98 (−1.98, 0.03)	−1.41 (−2.80, −0.03)*
Land surface temperature (LST), median, °C	−1.08 (−2.07, −0.08)*	−0.22 (−0.33, −0.12)***	0.09 (−0.20, 0.37)	−0.10 (−0.22, 0.02)	−1.48 (−3.00, 0.03)	−2.79 (−4.87, −0.71)**

*Note: Unit of analysis:* ABS Postal Areas (POAs) in Victoria, Australia. Cells show β (95% confidence interval) from spatial lag regression models fitted with a queen contiguity spatial weights matrix, which account for spatial autocorrelation between neighbouring postcodes. One model was fitted per outcome; all predictors listed were entered simultaneously and selected a priori. Outcomes are rates of self‐reported long‐term conditions per 1000 WRA from the 2021 Census. *β* is the change in the outcome rate (per 1000 WRA) for a one‐unit increase in the predictor, in the units shown: sociodemographic composition per one percentage point; median age per year; mortgage repayment, rent and household income per Australian dollar; IRSAD per one unit of score; and each environmental exposure per one unit of its listed measurement unit. *Statistical significance:* *p < 0.05; **p < 0.01; ***p < 0.001. Estimates whose confidence interval excludes zero are marked accordingly; unmarked estimates are not statistically significant at *p* < 0.05. Values shown as −0.00 or 0.00 are non‐zero estimates smaller in magnitude than 0.005. Exposure sources and averaging periods are as described in the notes to Table S1. Column‐based trace gases (CO, HCHO, O_3_, SO_2_, atmospheric water vapour) reflect total atmospheric column content rather than surface‐level exposure.

Abbreviations: ABS, Australian Bureau of Statistics; AUD, Australian dollars; CI, confidence interval; HCHO, formaldehyde; IRSAD, Index of Relative Socio‐economic Advantage and Disadvantage (a SEIFA index; higher scores indicate greater advantage); LST, land surface temperature; NDVI, Normalised Difference Vegetation Index (range −1 to +1; higher values indicate greater greenness); NO_2_, nitrogen dioxide; O_3_, ozone; PM_2.5_, fine particulate matter with an aerodynamic diameter < 2.5 μm; POA, Postal Area; ppb, parts per billion; SEIFA, Socio‐Economic Indexes for Areas; WRA, women of reproductive age (females aged 15–49 years).

Environmental exposures showed mixed patterns. Greenness was positively associated with asthma (*β* = 47.41; 95% CI: 0.82, 94.00). After adjustment for socioeconomic factors, PM_2.5_ was negatively associated with mental health burden (*β* = −4.37; 95% CI: −8.06, −0.68) and composite health outcomes burden (*β* = −6.00; 95% CI: −10.97, −1.03). Maximum and median land surface temperatures were negatively associated with cancer and composite outcomes.

In the stratified sensitivity analysis, some exposure‐health associations varied by urbanicity, while the socioeconomic gradient remained broadly consistent across strata. For example, the inverse association between PM2.5 and composite burden was confined to Regional Victoria and was absent in Metropolitan Melbourne. Because these analyses were exploratory and some strata were small, the findings are interpreted cautiously. Full stratum‐specific estimates are provided in the Supporting Information (Tables S2–S5).

### 3.4 Cluster Analysis of Metropolitan and Regional Postcodes

**FIGURE 4:**
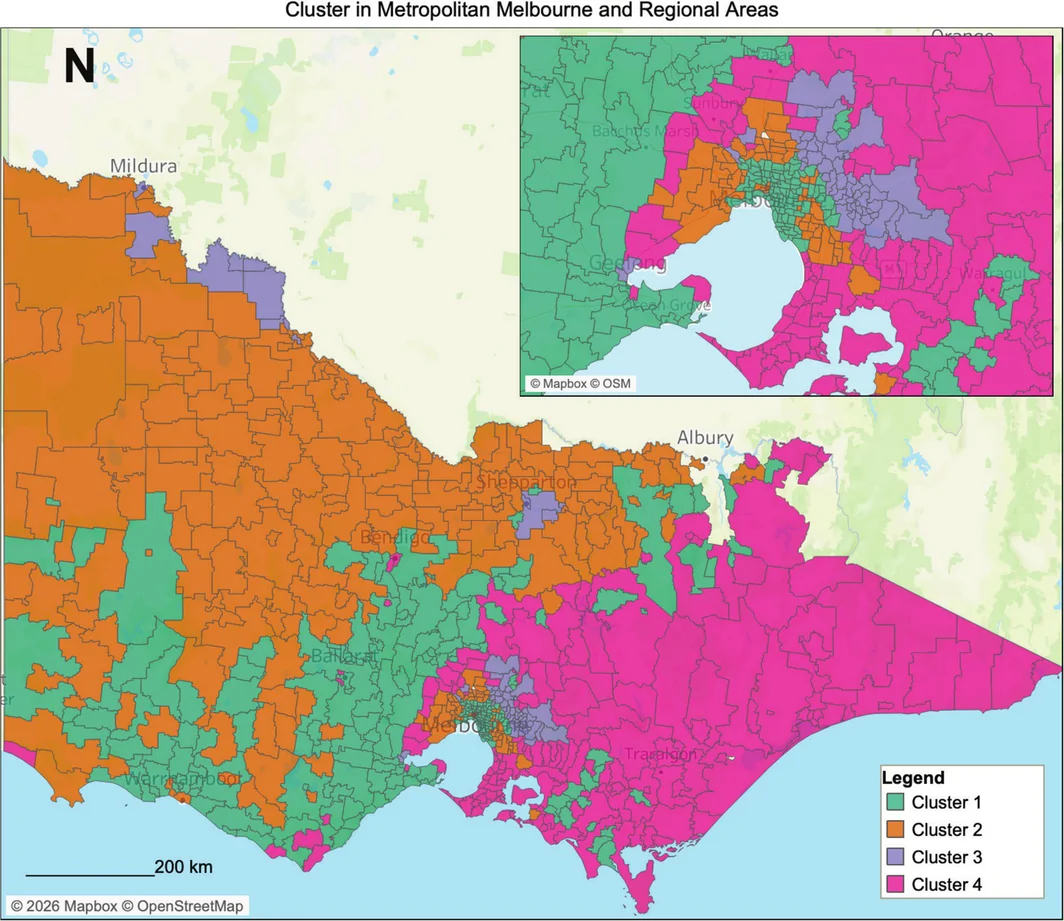
Clusters identified through K‐means by Victorian postcode. *Clusters in metropolitan Melbourne*, *Cluster 1*: Inner‐city, high‐SES, high pollution, lower disease burden affluent inner Melbourne suburbs with the highest NO_2_ and PM_2.5_ but strong socioeconomic conditions. Despite higher pollution, they show relatively lower chronic disease burden, suggesting strong social infrastructure buffers environmental risk. *Cluster 2*: Multicultural, younger, disadvantaged, low composite burden but high culturally diverse, socioeconomically disadvantaged suburbs with the highest proportion of overseas‐born residents. They have lower overall chronic disease burden but elevated diabetes rates, indicating metabolic vulnerability. *Cluster 3*: Older, affluent, low pollution, unexpectedly high chronic burden environmentally advantaged suburbs with low pollution and high SEIFA scores but an older population. Despite favourable environmental conditions, they show high asthma and mental health burden. *Cluster 4*: Peri‐urban, hottest areas, high chronic disease burden. Located on Melbourne's fringe, this cluster experiences the highest land surface temperatures and moderate socioeconomic disadvantage. They show the highest composite chronic disease burden, especially for asthma and mental health conditions. *Clusters in Regional Victoria*, *Cluster 1*: Older, moderate‐SES, low pollution, mid‐level chronic burden, low air pollution, moderate land surface temperature and older, predominantly Australian‐born populations. They show mid‐range chronic disease burden, with elevated asthma but relatively low diabetes. *Cluster 2*: Most disadvantaged, ageing, hottest areas with highest chronic burden, lowest SEIFA scores, lowest incomes and the highest land surface temperatures. It has the highest composite chronic disease burden and elevated asthma and mental health conditions, reflecting concentrated structural and environmental vulnerability. *Cluster 3*: Younger, diverse, hottest with extremely high diabetes rates, youngest population, highest cultural diversity and the highest temperatures. They exhibit **very high diabetes prevalence** but lower mental health and asthma burden, producing a mixed‐risk clinical profile. *Cluster 4*: Low‐income, older, high PM_2.5_ and high chronic burden consists of older, low‐income communities with the highest PM_2.5_ levels in regional Victoria. They show elevated mental health, cancer and cardio‐renal burden, contributing to a high overall chronic disease burden. *Inlet map is Metropolitan Melbourne.

Cluster analysis in Figure 4 identified four distinct spatial profiles in metropolitan Melbourne and regional Victoria. Cluster 4, comprising outer peri‐urban and fringe areas, had the highest burden of NCDs among WRA, including the highest rates of mental health conditions (273.4 per 1000), asthma and composite burden. These areas also recorded the highest land surface temperatures (median LST = 75.1) and elevated PM_2.5_ levels (17.2 μg/m^3^). Cluster 2 represented inner to middle‐ring diverse suburbs, characterised by high cultural diversity, low English proficiency and lower socioeconomic status (IRSAD = 5.5); this group recorded the highest diabetes prevalence (5.9 per 1000) and elevated long‐term illness rates. Cluster 1 included inner‐city, high‐income postcodes with strong SEIFA scores (IRSAD = 9.4) and higher education levels. Despite high NO_2_ and PM_2.5_ exposure, these areas had lower prevalence of diabetes and cardio‐renal disease but reported elevated asthma (6.0 per 1000) and mental health conditions (252.5 per 1000). Cluster 3 captured older, low‐density, environmentally advantaged suburbs with low pollution and high SEIFA, but had elevated mental health (267.9 per 1000) and cardio‐renal/stroke burden (152.3 per 1000), suggesting persistent chronic disease despite social advantage.

In regional Victoria, Cluster 3 encompassed remote and outer‐regional postcodes with the most adverse profile. These areas had the highest air pollutant concentrations (PM_2.5_ = 30.0 μg/m^3^), the lowest SEIFA scores (IRSAD = 2.9) and the highest diabetes prevalence (4.1 per 1000). Cluster 4 included lower‐income, mixed regional towns, with moderate pollution and high heat exposure (max LST = 18.2°C) and recorded the highest burden of cancer, cardio‐renal disease and composite outcomes. Cluster 2 comprised older, socioeconomically disadvantaged zones with the highest land surface temperatures and elevated asthma and mental health conditions. Cluster 1 captured advantaged inner‐regional areas, with older, Australian‐born populations, low cultural diversity, low pollution exposure and the lowest overall burden of disease.

## 4 Discussion

### 4.1 Principal Findings and Interpretation

Our study demonstrates marked spatial disparities in NCD burden among women of reproductive age (WRA) in Victoria. Asthma, diabetes, cardio‐renal conditions and mental health problems were disproportionately concentrated in low socioeconomic status postcodes. These findings are consistent with longstanding evidence that health is closely patterned by socioeconomic factors such as income, education, housing and healthcare access [20, 21].

Critically, this relationship is likely bidirectional. While socioeconomic deprivation increases the risk of developing NCDs, the onset of disease can itself lead to income loss, increased financial burden from healthcare costs and reduced productivity, thereby deepening socioeconomic deprivation over time. This creates a reinforcing cycle where health and economic vulnerability are inextricably linked, particularly for WRA who often bear disproportionate caregiving responsibilities.

Our findings confirm that environmental risks are present across Victoria, with elevated levels of NO_2_ and PM_2.5_ in central Melbourne and high LST in peri‐urban and regional areas. However, the health impact of these exposures appears to be significantly mitigated by socioeconomic advantage. This suggests that social factors including healthcare access, high‐quality housing and protective urban design (urban shading, building density and proximity to coastal airflow) can buffer populations against environmental risks. Therefore, while environmental risks are evident, health vulnerability is also substantially modified by these social factors, specially for those socioeconomically advantaged groups [22, 23].

Not all postcodes with elevated exposures to heat and air pollution reported high NCD burden, suggesting that vulnerability emerges not solely from environmental risk but from its interaction with social conditions. In peri‐urban and regional areas, socioeconomic deprivation often coincided with elevated land surface temperature and poorer access to services, producing clusters of compounded risk. Conversely, central Melbourne's lower disease burden, despite higher measured pollution in some pockets, may reflect the mediating role of social and built environment factors [24]. This pattern is consistent with recent research from Great Britain, which found that among more socioeconomically advantaged populations, health‐harming environmental exposures did not necessarily correspond to higher mortality, suggesting that greater socioeconomic resources may offset some of their adverse effects [25]. This supports our interpretation that high socioeconomic advantage buffers the harmful effects of environmental exposures in the Victorian context, through stronger social infrastructure and better access to healthcare, which can weaken the observable associations.

The sensitivity analysis suggests that pooled models should be interpreted cautiously because some exposure‐health associations appeared to vary across metropolitan, suburban and regional contexts. This does not change the main interpretation that socioeconomic disadvantage was the most consistent correlate of NCD burden among WRA, but it indicates that environmental associations may be context‐specific and should not be overgeneralised across Victoria.

### 4.2 Results in the Context of What Is Known

Findings from this study are strongly supported by the Victorian Population Health Survey (VPHS) 2022, which similarly reported consistent social gradients in the prevalence of chronic conditions such as obesity, Type 2 diabetes, asthma and psychological distress. Across multiple outcomes, the VPHS identified women who were financially stressed, lived in rental housing, had low educational attainment and household incomes below $40 000 as disproportionately affected—closely matching the geographic and socioeconomic clusters identified in our spatial analysis [26]. These parallels reinforce the interpretation that chronic disease risk among WRA in Victoria is driven more by entrenched socioeconomic deprivation than by environmental exposure alone, even under relatively low pollution conditions. The alignment between individual‐level VPHS indicators and our area‐level findings highlights the need for place‐based interventions that address the compounding effects of poverty, housing insecurity and psychosocial stress on women's health [26].

Previous research has established robust associations between air pollution, elevated temperature and chronic health outcomes [6, 27, 28]. However, in high‐income settings like Australia where many pollutants remain within regulatory thresholds [29, 30], health outcomes tend to reflect broader social contexts and the capacity to buffer or adapt to environmental risk [31, 32, 33, 34].

Our findings align with this pattern. While NO_2_ and PM_2.5_ levels were elevated in parts of Melbourne, ozone, SO_2_ and formaldehyde levels were low, especially in the CBD. As documented in environmental monitoring reports [29], inner‐urban areas often exhibit lower ozone levels, likely due to titration from high traffic emissions and SO_2_ remains negligible outside industrial zones [29, 30]. Likewise, formaldehyde is typically present in trace levels well below thresholds of concern. These relatively low pollutant levels may help explain the absence of strong environmental‐health gradients in some central metropolitan postcodes. However, in outer areas with less infrastructure and weaker service provision, even moderate environmental exposures appear to have greater health implications.

Our study contributes to a growing literature examining environmental risk among women of reproductive age beyond the perinatal period, an often‐overlooked group for non‐communicable disease (NCD) outcomes. Our findings align with the evidence that even low‐level environment exposures within regulatory limits can harm vulnerable populations [28, 35], particularly those with limited social and structural resources who faced elevated risk from relatively modest exposures like land surface temperature (LST) [22]. Our spatial analysis identifies distinct clusters of postcodes where WRA with a high burden of long‐term conditions are concentrated. In these communities, characterised by socioeconomic deprivation, low‐level environmental exposure may act as a critical tipping point, shaping vulnerability through cumulative social stressors over time.

### 4.3 Policy and Public Health Relevance

From an environmental justice perspective, disadvantaged peri‐urban and regional communities face disproportionate NCD burdens that are primarily driven by social disadvantage and are heightened by co‐occurring environmental risk. These disparities are evident under current conditions, raising concerns for future resilience. While this study examined air quality exposure levels mostly considered as under a safe threshold, any future intensification of climate‐related stressors, including extreme heat, is likely to exacerbate the health burden among structurally vulnerable WRA, demonstrating that compliance with regulatory limits alone is insufficient to protect especially populations with low adaptive capacity.

Public health interventions should prioritise regions where social disadvantage coincides with environmental exposure, particularly peri‐urban and regional postcodes. Interventions must go beyond traditional air quality management to address upstream determinants such as housing quality, energy efficiency, health service availability and culturally responsive care that proactively addresses the diagnostic and reporting barriers identified in culturally diverse communities [36, 37, 38]. Integrating place‐based, cross‐sectoral approaches will be essential to closing the health equity gap.

### 4.4 Research Directions

Future research should account for spatial and temporal dynamics in exposure and disease onset. Many chronic conditions in this study were self‐reported and may have developed before individuals moved to their current postcodes, raising concerns around exposure misclassification. Moreover, the environmental exposures were averaged over 4 years (2018–2021), a period that included both the 2019–2020 bushfires and COVID‐19 lockdowns—events that likely produced sharp but uneven changes in both air quality and access to care. These short‐term dynamics were not captured by the time‐averaged indicators.

Future studies should also explore the long‐term health impacts of extreme climate events, such as bushfires and floods, on the prevalence and progression of NCDs. While current evidence in Australia has identified the significant short‐term impacts of such environmental shocks on mental health [8], longitudinal research is required to determine how these events shape chronic physical and mental health trajectories over the life course.

More granular spatial data (e.g., Statistical area 1 or mesh block) and comprehensive residential history are needed to address these limitations. In addition, studies incorporating non‐linear exposure models, cumulative risk metrics and qualitative data from WRA in high‐risk areas would strengthen the evidence base for targeted interventions.

### 4.5 Strengths and Limitations

A key strength of this study is its focus on WRA, a population often underrepresented in environmental health surveillance and chronic disease research. By integrating multi‐source environmental, social and health data across Victoria, we were able to identify intersecting geographic vulnerabilities at scale. The use of spatial clustering, concentration indices and regression analysis offered a robust platform for uncovering these patterns.

Nonetheless, several limitations must be acknowledged. First, the associations observed at the postcode level may not reflect individual‐level risks, and our findings are susceptible to residual confounding. This likely explained some counterintuitive results, such as negative associations observed for formaldehyde and ozone, which may rise if these pollutants act as proxies for unmeasured urban advantages not fully captured by our socioeconomic adjustments. Similar methodological challenges have been noted in other national‐scale studies, where ‘health‐promoting’ amenities were unexpectedly associated with higher mortality due to complex co‐location of business density, traffic and socioeconomic self‐selection [25]. Similarly, the analysis of postcode level aggregates also presents challenges in interpreting correlations. For instance, the positive association of median rent with long‐term health conditions may reflect its role as an indicator for urban location and housing‐related stress, rather than being a direct risk factor itself. This demonstrates the difficulty of isolating effects of socioeconomic factors, which can sometimes operate in opposition to the overall advantage or disadvantage captured by a composite index like SEIFA‐IRSAD.

Furthermore, health outcomes were self‐reported and susceptible to recall bias and/or under‐reporting. Specifically, the lower reported prevalence of mental health disorders in culturally diverse areas (e.g., Metro Cluster 2 and Regional Cluster 3) may reflect recall bias, reluctance to self‐report due to stigma, or underdiagnosis, rather than a lower underlying disease burden. Diagnosis rates may also vary according to wealth and access to care, particularly in communities with poor healthcare access or cultural barriers to care. Consequently, because disadvantaged populations face greater barriers to diagnosis, the observed disparities likely understate the true extent of inequality. The lack of residential history limits our ability to match exposure timing to disease onset. Our cross‐sectional design captures area‐level conditions at a single point in time, meaning we cannot determine whether individuals developed their conditions while residing in their current area or prior to relocation. This is particularly relevant for chronic conditions with long latency periods, where cumulative environmental and socioeconomic exposures over the life course may be more salient than current residential context. Additionally, the use of postcode‐level data may mask differences in lifestyle and the nature of work (e.g., agricultural vs. office‐based) between regional towns and more remote settlements, which are often bundled together within the same administrative divisions. Environmental indicators were averaged over a four‐year window that included highly atypical periods, such as the bushfires and pandemic lockdowns. This approach likely smoothed over important short‐term spikes in exposure that may have driven health effects, particularly among more vulnerable groups.

Taken together, these limitations suggest that the observed disparities may understate the true extent of health inequality and environmental vulnerability among WRA. Continued investment in linked, individual‐level spatial health data is needed to more accurately quantify these risks.

## 5 Conclusion

Our study reveals that NCD burden among WRA in Victoria is associated with the interaction of structural disadvantage with clustering of environmental exposures. Importantly, this relationship may also be bi‐directional; while disadvantage increases disease risk, the onset of NCD can also lead to income loss and increased financial toxicity, potentially deepening disadvantage over time. These disparities persist even under environmental conditions deemed acceptable by current regulatory standards. Without targeted, equity‐oriented intervention, future environmental shocks are likely to compound existing health burdens. Preventing the widening of health disparities among WRA will require proactive, place‐based policies that address both environmental conditions and the social systems that influence how and for whom those conditions become harmful.

## Funding

The study was funded by the Norman Beischer Medical Research Foundation Innovation Grant, the University of Melbourne Early Career Research Grant and the University of Melbourne Climate Futures, Climate Research Accelerator 2026. MM and SM receive salary support from Generation Victoria (GenV) of the Murdoch Children's Research Institute. MM also received salary support from Mercy Perinatal. MM is supported by the University of Melbourne Department of Obstetrics, Gynaecology and Newborn Health Early Career Research Fellowship and 2026 Climate Change Accelerator Grant of the University of Melbourne. SM is supported by a FAIR Fellowship 2024 Award administered by veski for the Victorian Health and Medical Research Workforce Action Plan on behalf of the Victorian Government. Funding for the Award has been provided by the Victorian Department of Jobs, Skills, Industry and Regions. Research at the Murdoch Children's Research Institute is supported by the Victorian Government's Operational Infrastructure Program.

## Ethics Statement

Ethical approval for this study was obtained from the Human Research Ethics Committee of Mercy Health (Ref. no 2023‐044).

## Conflicts of Interest

The authors declare no conflicts of interest.

## Supporting information


**Table S1:** ISLE‐ReSt reporting standards.
**Table S2:** Sensitivity analysis: geospatial (spatial lag) regression restricted to Metropolitan Melbourne (*n* = 263 postcodes).
**Table S3:** Sensitivity analysis: geospatial (spatial lag) regression restricted to Regional Victoria (*n* = 431 postcodes).
**Table S4:** Secondary sensitivity analysis restricted to inner‐city metropolitan postcodes (centroid within 10 km of the Melbourne CBD; *n* = 53).
**Table S5:** Secondary sensitivity analysis restricted to suburban metropolitan postcodes (more than 10 km from the CBD; *n* = 210). Regional Victoria estimates are given in Table S3.

## Data Availability

The datasets analysed in this study include publicly available data from the Australian Bureau of Statistics (ABS) 2021 Census and environmental datasets obtained from catalogues hosted on Google Earth Engine. Specific dataset names and access details are provided in the manuscript. All data are publicly available from the original sources through their respective repositories.
