# Supplementary material for: A Geospatial Linkage Analysis of Environmental Exposures, Socioeconomic Profile and Health Outcomes Among Women of Reproductive Age in Victoria, Australia
Source: Health Promot J Austr. 2026 Aug 28;37(4):e70241. doi: 10.1002/hpja.70241 (PMC13523080; doi:10.1002/hpja.70241)
Supplement: Supplementary file 1 [file HPJA-37-0-s001.docx]

**Supplementary Files**

**Detailed Methodologies:**

**PM_2.5_**

**Fine particulate matter (PM₂.₅) concentrations** were obtained from the CSIRO Chemical Transport Model (CTM). [1] This model uses emissions inventories, meteorological inputs, and chemical transport simulations to estimate surface-level concentrations of PM₂.₅ across Australia. Outputs are provided on a high-resolution grid and have been validated against ground-based monitoring networks. For our study, we used multi-year average annual PM₂.₅ estimates (µg/m³) and aggregated them to postcode boundaries in Victoria.

**NO_2_**

**Nitrogen dioxide (NO₂) concentrations** were obtained from the national land-use regression (LUR) model developed by Knibbs et al. (2014).[2] This model was built using annual average NO₂ data from 160 monitoring sites across Australia, combined with geographic predictors including satellite-derived tropospheric NO₂ (OMI sensor), roadway density and traffic, population density and urban land use, elevation, and climatic variables (temperature and rainfall). For our study, we used the published surface-level annual NO₂ estimates (µg/m³) and aggregated them to postcode boundaries across Victoria.

**Land Surface Temperature**

We estimated annual Land Surface Temperature (LST) for Victoria, Australia, from 2018 to 2023 using the Google Earth Engine (GEE) cloud-computing platform. We used data from the Landsat 8 Operational Land Imager/Thermal Infrared Sensor (OLI/TIRS) Collection 2, Level 2 Surface Reflectance and Surface Temperature product (LANDSAT/LC08/C02/T1_L2), which provides atmospherically corrected surface reflectance (30 m resolution) and surface temperature bands derived from thermal infrared measurements. Images were filtered to the Victoria state boundary and constrained to yearly time windows (January 1 to December 31). To minimise contamination, cloud and cloud-shadow pixels were masked using the Quality Assessment (QA_PIXEL) band. A median composite was generated for each year to represent annual conditions.

**Normalised Difference Vegetation Index (NDVI)**

NDVI was calculated within GEE from the surface reflectance bands (Band 5: near-infrared, Band 4: red) rather than using pre-computed indices, to derive fractional vegetation cover (FVC). FVC values were then used to estimate pixel-level surface emissivity following a standard empirical relationship (ε = 0.986 + 0.004 × FVC). Thermal Band 10 (ST_B10) was converted to top-of-atmosphere brightness temperature (Kelvin) using radiometric rescaling factors (0.00341802 × DN + 149.0). LST (°C) was subsequently derived using the radiative transfer equation corrected for emissivity.

Resulting LST rasters were clipped to Victoria, and summary statistics (mean, median, minimum, maximum) were extracted for each postal area polygon using GEE’s reduceRegions function. as CSV tables for subsequent analyses.

**CO_2_, SO_2_, HCHO, O_3_**

We extracted atmospheric columnar gas concentrations for carbon monoxide (CO), sulphur dioxide (SO₂), formaldehyde (CH_2_O), and ozone (O₃) from the Sentinel-5 Precursor (Sentinel-5P) satellite, which carries the TROPOspheric Monitoring Instrument (TROPOMI). Sentinel-5P provides global daily observations of trace gases at a native resolution of ~3.5 × 7 km, improved to ~3.5 × 5.5 km from August 2019 onwards, with products available via the Google Earth Engine data catalogues.[3] Monthly means were calculated for the period 2019–2023, aggregated to annual means, and spatially averaged within postcode polygons to generate annual postcode-level exposure estimates.

# Table 1: ISLE-ReSt reporting standards

| Component | Item No | Recommendation | Page/Line number |
| --- | --- | --- | --- |
| Title | 1 | Indicate the primary exposure variable(s) and main outcome variable(s) | 1 |
| Abstract | 2 | Provide an informative and balanced summary of objectives, methods (including study design, exposure and outcome variables), results, and conclusions | 1, 2 |
| Introduction |  |  |  |
| Background/Rationale | 3 | Explain the scientific background and rationale for the investigation, including conceptual links between environmental and health variables | 2,3 |
| Objectives | 4 | State specific objectives, including pre-specified hypotheses | 3 |
| Methods |  |  |  |
| Study Design | 5 | Present a full description of study design, with rationale for the spatial scale of exposure | 3 |
| Setting | 6 | Describe the setting, locations, and relevant dates (e.g., recruitment, exposure, follow-up) | 4 |
| Participants/Sample Size | 7 | Give eligibility criteria, methods of selection, follow-up, and linkages to spatial locations | 4 |
| Variables | 8 | Clearly define all outcomes, exposures, predictors, confounders, and effect modifiers | 4,5 |
| Health Data Sources/Measurement | 9 | Provide sources of data and details of methods of assessment | 3 |
| Bias | 10 | Describe efforts to address potential sources of bias | 4 |
| Quantitative Variables | 11 | Explain how quantitative variables were handled in the analyses | 4 |
| Spatial Data | 12 | Provide data sources, spatial resolution, and processing methods for GIS, RS, GPS, or sensor data | 4 |
| Spatial Methods | 13 | Describe methods and software used for spatial analysis, including preprocessing of spatial data | 5 |
| Statistical Methods | 14 | Describe statistical methods used, including how spatial autocorrelation was addressed | 4,5 |
| Results |  |  |  |
| Participants | 15 | Report numbers of individuals at each stage of the study, possibly using a flow diagram | 6 |
| Descriptive Data | 16 | Provide characteristics of participants and exposure information | 6,7 |
| Outcome Data | 17 | Report numbers of outcome events or summary measures over time | 7 |
| Main Results | 18 | Provide unadjusted and adjusted estimates with their precision | 7 |
| Other Analyses | 19 | Report other analyses like subgroup or mediation analysis, and spatial autocorrelation diagnostics | 6 |
| Discussion |  |  |  |
| Key Results | 20 | Summarize key results in relation to the objectives | 8 |
| Interpretation | 21 | Provide an overall interpretation, considering study limitations and evidence from similar studies | 9 |
| Limitations | 22 | Describe limitations such as exposure misclassification, geographic context problems, or biases | 11 |
| Generalizability | 23 | Discuss the external validity of the study results | 9,10 |
| Other Information |  |  |  |
| Source of Funding | 24 | Provide the source of funding and the role of funders | Title Page |
| Conflict of Interest | 25 | Describe potential conflicts of interest | N/A |
| Data Sharing Statement | 26 | Provide details about which data can be shared and how to access it | 11 |

**Glossary of Research Terms**

This glossary aims to assist readers in understanding commonly used terms and concepts when reading the research. The definitions have been adapted from the cited sources.

1. Population characteristics:

- First Nations
- Born in Australia
- Born elsewhere refers to the population group who were born outside Australia. The top 5 countries include England, China, India, Philippines, and New Zealand.
- English-only speakers
- Other language speakers refer to the population who used other language than English in their homes including Mandarin, Arabic, Vietnamese, Cantonese, and Punjabi.
- Australian citizens

2. Socioeconomic indicators:

- Index of Relative Advantage and Disadvantage (IRSAD) summarizes the economic and social conditions within a geographic area. A high IRSAD score reflects greater socioeconomic advantage and less disadvantage in an area, while a low score indicates the opposite.
- Socio-Economic Indexes for Areas (SEIFA) is an area-based ranking system in Australia according to relative socio-economic advantage and disadvantage, evaluated in five-yearly Census. It consists of four indexes: Index of Relative Socio-Economic Disadvantage (IRSD), IRSAD, Index of Education and Occupation (IEO), and IER.

3. Environmental exposures:

- Normalized Difference Vegetation Index (NDVI) is a measure of live green vegetation that quantifies the difference between how plants absorb visible red light and reflect near-infrared light. It is calculated from satellite data and provides an indicator of vegetation density, greenness, and photosynthetic capacity.
- Land Surface Temperature is the radiometric "skin" temperature of the Earth's surface, derived from measured thermal radiance.

**Supplementary Methods. Sensitivity analysis: stratification by urbanicity**

We conducted a sensitivity analysis to assess whether the exposure vulnerability associations were consistent across settings. Rather than adjusting for metropolitan status within a single pooled model, we refitted each spatial simultaneous autoregressive (lag) model separately within urbanicity strata, using the same variables, outcomes, and layout as the main-text regression table (Table 2). In the primary analysis we stratified postcodes into Metropolitan Melbourne (n = 263; Supplementary Table 2) and Regional Victoria (n = 431; Supplementary Table 3). In a secondary analysis we further divided the metropolitan postcodes into an inner-city tier, defined as postcodes whose centroid lay within 10 km of the Melbourne central business district (postcode 3000; n = 53; Supplementary Table 4), and a suburban tier (n = 210; Supplementary Table 5); the Regional Victoria estimates for this three-tier analysis are identical to those in Supplementary Table 3. Postcode centroids were projected to GDA94 / Vicgrid (EPSG:3111) to compute these distances. Within each stratum we rebuilt the queen contiguity spatial weights matrix and refitted each model adjusting for socioeconomic disadvantage (IRSAD), the proportion of women of reproductive age, and median age; the metropolitan indicator was omitted because it is constant within a stratum, and any exposure that was itself an adjuster was dropped from the adjustment set for its own model. Models were fitted with the spdep and spatialreg packages in R.

**Table 2. Sensitivity analysis: geospatial (spatial lag) regression restricted to Metropolitan Melbourne (n = 263 postcodes).**

*Each cell shows the exposure coefficient and (95% confidence interval). * p < 0.05; ** p < 0.01; *** p < 0.0001. NE = not estimated (model did not converge or too few postcodes).*

| Exposure | Asthma | Cancer | Diabetes | Cardiorenal and Stroke | Mental Health Conditions | Composite Long Term |
| --- | --- | --- | --- | --- | --- | --- |
| Proportion |  |  |  |  |  |  |
| First nations | 23.75  (18.36, 29.14)*** | -0.10  (-0.79, 0.59) | -0.85  (-2.10, 0.40) | -0.00  (-0.78, 0.78) | 27.18  (18.71, 35.66)*** | 50.79  (37.05, 64.52)*** |
| Born in Australia | 1.55  (1.33, 1.77)*** | 0.05  (0.02, 0.08)** | -0.00  (-0.05, 0.05) | 0.03  (-0.00, 0.06) | 2.34  (1.99, 2.70)*** | 4.15  (3.59, 4.70)*** |
| Born elsewhere | -1.51  (-1.79, -1.24)*** | -0.03  (-0.06, 0.00) | 0.04  (-0.01, 0.10) | -0.01  (-0.04, 0.03) | -2.27  (-2.71, -1.82)*** | -3.92  (-4.63, -3.20)*** |
| Speaking English only | 1.10  (0.92, 1.28)*** | 0.03  (0.01, 0.05)** | -0.00  (-0.04, 0.04) | 0.02  (-0.01, 0.04) | 1.81  (1.52, 2.10)*** | 3.13  (2.68, 3.58)*** |
| Speaks other foreign language | -1.00  (-1.20, -0.80)*** | -0.02  (-0.04, 0.00) | 0.02  (-0.02, 0.07) | -0.01  (-0.03, 0.02) | -1.66  (-1.98, -1.33)*** | -2.80  (-3.31, -2.28)*** |
| Australia Citizen | 1.80  (1.50, 2.10)*** | 0.10  (0.06, 0.14)*** | 0.06  (-0.02, 0.14) | 0.06  (0.01, 0.11)* | 2.57  (2.09, 3.04)*** | 4.71  (3.97, 5.45)*** |
| Selected median |  |  |  |  |  |  |
| Person age | 0.96  (0.02, 1.90)* | -0.08  (-0.20, 0.03) | -0.22  (-0.43, -0.01)* | 0.00  (-0.12, 0.13) | 2.04  (0.61, 3.47)** | 3.03  (0.69, 5.37)* |
| Mortgage repayment | -0.01  (-0.02, -0.00)** | 0.00  (-0.00, 0.00) | -0.00  (-0.00, 0.00) | 0.00  (-0.00, 0.00) | -0.02  (-0.04, -0.01)** | -0.04  (-0.06, -0.01)** |
| Median rent | 0.00  (-0.06, 0.06) | 0.01  (0.01, 0.02)*** | 0.01  (-0.00, 0.03) | 0.01  (0.00, 0.02)** | -0.03  (-0.12, 0.06) | 0.00  (-0.15, 0.15) |
| Total household income | 0.02  (0.01, 0.03)** | 0.00  (0.00, 0.00)** | 0.00  (-0.00, 0.00) | 0.00  (-0.00, 0.00) | 0.01  (-0.00, 0.03) | 0.03  (0.01, 0.06)* |
| SEIFA |  |  |  |  |  |  |
| IRSAD | -1.93  (-3.20, -0.67)** | 0.04  (-0.11, 0.19) | -1.02  (-1.32, -0.73)*** | -0.55  (-0.73, -0.37)*** | -2.31  (-4.19, -0.43)* | -5.14  (-8.27, -2.01)** |
| External environment |  |  |  |  |  |  |
| NDVI | 79.49  (30.12, 128.87)** | -4.99  (-10.81, 0.83) | -2.71  (-13.23, 7.81) | -7.59  (-14.21, -0.98)* | 142.98  (68.64, 217.32)** | 221.19  (99.30, 343.07)** |
| Nitrogen dioxide | -2.27  (-3.79, -0.75)** | 0.18  (-0.00, 0.36) | -0.11  (-0.43, 0.22) | 0.16  (-0.04, 0.36) | -2.25  (-4.48, -0.02)* | -4.19  (-7.89, -0.48)* |
| Atmospheric water vapour | -0.02  (-0.12, 0.09) | 0.02  (0.01, 0.03)** | 0.04  (0.02, 0.07)** | 0.01  (0.00, 0.03)* | -0.13  (-0.28, 0.03) | -0.12  (-0.38, 0.13) |
| Formaldehyde | 10.96  (-61.99, 83.90) | -10.10  (-18.86, -1.34)* | -0.86  (-16.62, 14.90) | -10.50  (-20.27, -0.72)* | -9.21  (-117.89, 99.46) | -20.27  (-199.57, 159.04) |
| Ozone | 43.41  (-6.41, 93.22) | -2.42  (-8.30, 3.46) | 2.77  (-7.90, 13.44) | 4.26  (-2.40, 10.93) | 41.50  (-32.53, 115.53) | 83.02  (-39.51, 205.54) |
| PM2.5 | -2.37  (-5.34, 0.60) | 0.42  (0.06, 0.77)* | 0.30  (-0.33, 0.93) | 0.39  (-0.01, 0.78) | -1.73  (-6.12, 2.66) | -2.88  (-10.14, 4.38) |
| Maximum LST | 0.32  (-0.63, 1.27) | -0.01  (-0.12, 0.11) | 0.17  (-0.04, 0.37) | -0.05  (-0.17, 0.08) | -0.56  (-1.97, 0.86) | -0.38  (-2.72, 1.95) |
| Median LST | -0.91  (-2.74, 0.91) | 0.06  (-0.16, 0.28) | 0.80  (0.39, 1.21)** | 0.08  (-0.17, 0.33) | -2.68  (-5.44, 0.08) | -3.48  (-8.00, 1.04) |

**Table 3. Sensitivity analysis: geospatial (spatial lag) regression restricted to Regional Victoria (n = 431 postcodes).**

*Each cell shows the exposure coefficient and (95% confidence interval). * p < 0.05; ** p < 0.01; *** p < 0.0001. NE = not estimated (model did not converge or too few postcodes). Variables, outcome columns, and layout match Table 2 of the main manuscript.*

| Exposure | Asthma | Cancer | Diabetes | Cardiorenal and Stroke | Mental Health Conditions | Composite Long Term |
| --- | --- | --- | --- | --- | --- | --- |
| Proportion |  |  |  |  |  |  |
| First nations | -2.11  (-6.13, 1.92) | -0.21  (-0.65, 0.23) | -0.44  (-1.65, 0.78) | -0.30  (-0.79, 0.19) | -0.27  (-6.34, 5.79) | -3.28  (-11.34, 4.79) |
| Born in Australia | 1.30  (0.30, 2.29)* | 0.00  (-0.11, 0.11) | -0.28  (-0.58, 0.02) | -0.01  (-0.14, 0.11) | 1.75  (0.26, 3.25)* | 2.76  (0.77, 4.75)** |
| Born elsewhere | 0.29  (-0.90, 1.47) | 0.07  (-0.06, 0.20) | 0.38  (0.03, 0.74)* | 0.09  (-0.05, 0.24) | 0.88  (-0.89, 2.66) | 1.66  (-0.70, 4.02) |
| Speaking English only | 2.15  (1.18, 3.11)*** | 0.05  (-0.06, 0.16) | -0.18  (-0.48, 0.11) | 0.04  (-0.08, 0.16) | 3.16  (1.71, 4.61)*** | 5.19  (3.29, 7.09)*** |
| Speaks other foreign language | -1.51  (-2.79, -0.22)* | -0.05  (-0.19, 0.10) | 0.36  (-0.02, 0.75) | -0.03  (-0.19, 0.12) | -2.59  (-4.52, -0.66)** | -3.83  (-6.39, -1.27)** |
| Australia Citizen | 2.50  (1.37, 3.63)*** | 0.06  (-0.07, 0.19) | -0.29  (-0.64, 0.05) | 0.05  (-0.09, 0.19) | 2.90  (1.19, 4.61)** | 5.16  (2.92, 7.40)*** |
| Selected median |  |  |  |  |  |  |
| Person age | -3.01  (-4.46, -1.57)*** | -0.13  (-0.29, 0.03) | -0.49  (-0.92, -0.06)* | -0.14  (-0.32, 0.03) | -1.62  (-3.78, 0.55) | -5.38  (-8.26, -2.50)** |
| Mortgage repayment | 0.02  (0.00, 0.03)* | 0.00  (0.00, 0.01)*** | 0.00  (-0.00, 0.01) | 0.00  (0.00, 0.01)** | 0.04  (0.01, 0.06)** | 0.06  (0.03, 0.09)*** |
| Median rent | 0.14  (0.06, 0.22)** | 0.03  (0.02, 0.04)*** | 0.03  (0.01, 0.06)** | 0.02  (0.01, 0.03)** | 0.28  (0.16, 0.39)*** | 0.49  (0.34, 0.64)*** |
| Total household income | 0.01  (-0.02, 0.04) | -0.00  (-0.00, 0.00) | 0.01  (-0.00, 0.01) | -0.00  (-0.00, 0.00) | 0.03  (-0.01, 0.07) | 0.04  (-0.01, 0.10) |
| SEIFA |  |  |  |  |  |  |
| IRSAD | -8.20  (-10.55, -5.85)*** | -0.18  (-0.44, 0.07) | -1.28  (-1.99, -0.58)** | -0.57  (-0.85, -0.29)*** | -11.15  (-14.68, -7.62)*** | -21.36  (-26.10, -16.61)*** |
| External environment |  |  |  |  |  |  |
| NDVI | 57.35  (-11.20, 125.90) | 13.39  (5.82, 20.96)** | -1.69  (-22.45, 19.07) | 6.13  (-2.25, 14.50) | 35.29  (-68.09, 138.68) | 110.54  (-26.65, 247.72) |
| Nitrogen dioxide | 3.76  (-2.61, 10.13) | 1.00  (0.30, 1.70)** | 0.14  (-1.78, 2.06) | 1.59  (0.80, 2.37)*** | 5.76  (-3.83, 15.34) | 11.85  (-0.86, 24.56) |
| Atmospheric water vapour | -0.04  (-0.11, 0.04) | -0.01  (-0.01, 0.00) | -0.01  (-0.03, 0.01) | 0.00  (-0.01, 0.01) | -0.14  (-0.26, -0.03)* | -0.19  (-0.34, -0.04)* |
| Formaldehyde | 17.58  (-57.50, 92.66) | -9.07  (-17.31, -0.83)* | -4.36  (-26.99, 18.27) | -8.91  (-18.08, 0.26) | 25.43  (-87.44, 138.30) | 21.22  (-127.82, 170.26) |
| Ozone | -43.24  (-85.42, -1.07)* | -6.78  (-11.43, -2.13)** | -1.72  (-14.49, 11.06) | -4.48  (-9.64, 0.67) | -41.53  (-105.11, 22.05) | -96.23  (-180.27, -12.20)* |
| PM2.5 | -2.79  (-6.16, 0.57) | 0.02  (-0.35, 0.38) | -0.11  (-1.12, 0.90) | 0.22  (-0.19, 0.63) | -9.70  (-14.77, -4.63)** | -11.50  (-18.04, -4.96)** |
| Maximum LST | -0.87  (-1.70, -0.04)* | -0.12  (-0.21, -0.03)* | 0.18  (-0.07, 0.43) | -0.08  (-0.18, 0.02) | -1.32  (-2.57, -0.08)* | -2.19  (-3.85, -0.54)** |
| Median LST | -1.58  (-2.80, -0.37)* | -0.27  (-0.40, -0.13)*** | 0.01  (-0.36, 0.38) | -0.12  (-0.27, 0.03) | -1.92  (-3.75, -0.08)* | -3.85  (-6.27, -1.43)** |

**Table 4. Secondary sensitivity analysis restricted to inner-city metropolitan postcodes (centroid within 10 km of the Melbourne CBD; n = 53).**

*Each cell shows the exposure coefficient and (95% confidence interval). * p < 0.05; ** p < 0.01; *** p < 0.0001. NE = not estimated (model did not converge or too few postcodes). Variables, outcome columns, and layout match Table 2 of the main manuscript.*

| Exposure | Asthma | Cancer | Diabetes | Cardiorenal and Stroke | Mental Health Conditions | Composite Long Term |
| --- | --- | --- | --- | --- | --- | --- |
| Proportion |  |  |  |  |  |  |
| First nations | 30.21  (15.09, 45.33)*** | 3.16  (0.74, 5.58)* | 1.17  (-1.52, 3.86) | 0.50  (-1.68, 2.69) | 73.94  (40.05, 107.84)*** | 106.71  (58.34, 155.08)*** |
| Born in Australia | 1.74  (1.45, 2.02)*** | 0.15  (0.09, 0.21)*** | -0.02  (-0.09, 0.05) | 0.02  (-0.04, 0.07) | 3.61  (2.79, 4.43)*** | 5.54  (4.50, 6.58)*** |
| Born elsewhere | -1.78  (-2.09, -1.47)*** | -0.16  (-0.23, -0.10)*** | 0.02  (-0.05, 0.10) | -0.02  (-0.08, 0.04) | -3.72  (-4.60, -2.84)*** | -5.69  (-6.81, -4.57)*** |
| Speaking English only | 1.29  (1.01, 1.57)*** | 0.13  (0.07, 0.18)*** | -0.04  (-0.10, 0.03) | 0.01  (-0.04, 0.06) | 2.76  (2.04, 3.49)*** | 4.15  (3.19, 5.11)*** |
| Speaks other foreign language | -1.26  (-1.56, -0.96)*** | -0.13  (-0.18, -0.07)*** | 0.03  (-0.03, 0.10) | -0.01  (-0.07, 0.04) | -2.72  (-3.48, -1.95)*** | -4.07  (-5.09, -3.05)*** |
| Australia Citizen | 1.73  (1.40, 2.05)*** | 0.16  (0.10, 0.22)*** | -0.02  (-0.09, 0.05) | 0.01  (-0.05, 0.07) | 3.68  (2.81, 4.55)*** | 5.60  (4.46, 6.73)*** |
| Selected median |  |  |  |  |  |  |
| Person age | -2.09  (-3.88, -0.30)* | -0.02  (-0.30, 0.26) | -0.34  (-0.64, -0.05)* | 0.25  (0.00, 0.49)* | -1.99  (-6.11, 2.13) | -3.97  (-9.82, 1.87) |
| Mortgage repayment | -0.01  (-0.02, 0.01) | -0.00  (-0.00, 0.00) | -0.00  (-0.00, 0.00) | -0.00  (-0.00, 0.00) | -0.01  (-0.05, 0.02) | -0.03  (-0.07, 0.02) |
| Median rent | 0.02  (-0.07, 0.10) | -0.00  (-0.02, 0.01) | -0.01  (-0.03, 0.00) | 0.01  (-0.00, 0.02) | 0.03  (-0.17, 0.23) | 0.05  (-0.24, 0.33) |
| Total household income | 0.01  (-0.01, 0.03) | 0.00  (-0.00, 0.00) | 0.00  (-0.00, 0.00) | 0.00  (-0.00, 0.00) | 0.02  (-0.03, 0.07) | 0.04  (-0.03, 0.11) |
| SEIFA |  |  |  |  |  |  |
| IRSAD | 2.02  (-0.63, 4.66) | 0.22  (-0.20, 0.64) | -0.77  (-1.22, -0.32)** | -0.76  (-1.12, -0.40)*** | 5.26  (-0.90, 11.43) | 6.26  (-2.47, 14.98) |
| External environment |  |  |  |  |  |  |
| NDVI | -29.93  (-99.00, 39.15) | -3.10  (-14.11, 7.92) | -1.72  (-13.24, 9.81) | -10.97  (-20.14, -1.80)* | -83.70  (-244.54, 77.14) | -127.50  (-354.53, 99.54) |
| Nitrogen dioxide | -0.45  (-3.02, 2.11) | 0.04  (-0.36, 0.43) | -0.14  (-0.57, 0.28) | 0.34  (0.01, 0.67)* | -0.14  (-5.99, 5.71) | 0.19  (-8.10, 8.48) |
| Atmospheric water vapour | -0.06  (-0.42, 0.31) | -0.04  (-0.09, 0.02) | 0.02  (-0.04, 0.08) | -0.01  (-0.06, 0.03) | 0.04  (-0.80, 0.87) | -0.02  (-1.21, 1.17) |
| Formaldehyde | 9.15  (-194.47, 212.77) | 12.34  (-19.54, 44.22) | -6.13  (-39.71, 27.44) | -1.43  (-28.86, 26.00) | 112.10  (-361.72, 585.93) | 106.64  (-564.17, 777.45) |
| Ozone | -79.67  (-183.76, 24.41) | -3.17  (-19.51, 13.18) | -16.95  (-33.60, -0.30)* | 5.57  (-8.31, 19.45) | -158.08  (-396.70, 80.54) | -246.88  (-586.02, 92.25) |
| PM2.5 | -2.60  (-10.89, 5.69) | -0.86  (-2.12, 0.41) | -0.34  (-1.77, 1.08) | -0.41  (-1.50, 0.69) | -1.79  (-20.52, 16.95) | -3.88  (-30.53, 22.78) |
| Maximum LST | -0.93  (-3.86, 2.00) | -0.12  (-0.58, 0.34) | 0.62  (0.14, 1.09)* | 0.19  (-0.20, 0.59) | -4.55  (-11.40, 2.30) | -5.11  (-14.78, 4.56) |
| Median LST | 0.85  (-3.60, 5.31) | 0.18  (-0.52, 0.88) | 0.41  (-0.33, 1.15) | -0.38  (-0.98, 0.22) | -0.92  (-11.26, 9.42) | -0.15  (-14.79, 14.49) |

**Table 5. Secondary sensitivity analysis restricted to suburban metropolitan postcodes (more than 10 km from the CBD; n = 210). Regional Victoria estimates are given in Supplementary Table 3.**

*Each cell shows the exposure coefficient and (95% confidence interval). * p < 0.05; ** p < 0.01; *** p < 0.0001. NE = not estimated (model did not converge or too few postcodes). Variables, outcome columns, and layout match Table 2 of the main manuscript.*

| Exposure | Asthma | Cancer | Diabetes | Cardiorenal and Stroke | Mental Health Conditions | Composite Long Term |
| --- | --- | --- | --- | --- | --- | --- |
| Proportion |  |  |  |  |  |  |
| First nations | 25.37  (19.69, 31.06)*** | -0.16  (-0.92, 0.59) | -0.54  (-1.94, 0.86) | 0.23  (-0.62, 1.09) | 28.45  (19.69, 37.21)*** | 54.17  (40.02, 68.33)*** |
| Born in Australia | 1.53  (1.29, 1.78)*** | 0.04  (0.01, 0.07)* | 0.01  (-0.05, 0.07) | 0.04  (-0.00, 0.07) | 2.26  (1.88, 2.63)*** | 4.05  (3.47, 4.64)*** |
| Born elsewhere | -1.66  (-1.96, -1.36)*** | -0.02  (-0.06, 0.02) | 0.02  (-0.05, 0.09) | -0.02  (-0.07, 0.02) | -2.36  (-2.83, -1.89)*** | -4.19  (-4.94, -3.44)*** |
| Speaking English only | 1.17  (0.98, 1.36)*** | 0.03  (0.00, 0.05)* | 0.01  (-0.03, 0.06) | 0.03  (0.00, 0.06)* | 1.94  (1.65, 2.24)*** | 3.40  (2.96, 3.85)*** |
| Speaks other foreign language | -1.19  (-1.41, -0.98)*** | -0.02  (-0.04, 0.01) | 0.00  (-0.05, 0.05) | -0.02  (-0.05, 0.01) | -1.94  (-2.28, -1.60)*** | -3.37  (-3.89, -2.85)*** |
| Australia Citizen | 1.77  (1.40, 2.13)*** | 0.09  (0.04, 0.14)** | 0.07  (-0.03, 0.16) | 0.06  (0.01, 0.12)* | 2.32  (1.77, 2.87)*** | 4.40  (3.53, 5.27)*** |
| Selected median |  |  |  |  |  |  |
| Person age | 2.24  (0.79, 3.69)** | -0.07  (-0.24, 0.11) | 0.15  (-0.17, 0.46) | 0.15  (-0.04, 0.35) | 3.99  (1.89, 6.10)** | 6.58  (3.09, 10.06)** |
| Mortgage repayment | -0.02  (-0.03, -0.01)** | 0.00  (-0.00, 0.00) | -0.00  (-0.00, 0.00) | 0.00  (-0.00, 0.00) | -0.04  (-0.06, -0.02)*** | -0.06  (-0.09, -0.03)*** |
| Median rent | -0.02  (-0.10, 0.05) | 0.02  (0.01, 0.02)** | 0.01  (-0.01, 0.03) | 0.01  (-0.00, 0.02) | -0.07  (-0.18, 0.03) | -0.07  (-0.24, 0.10) |
| Total household income | 0.02  (0.00, 0.03)* | 0.00  (0.00, 0.00)* | 0.00  (-0.00, 0.00) | -0.00  (-0.00, 0.00) | 0.01  (-0.01, 0.03) | 0.03  (-0.00, 0.06) |
| SEIFA |  |  |  |  |  |  |
| IRSAD | -2.20  (-3.65, -0.74)** | -0.00  (-0.17, 0.17) | -0.98  (-1.31, -0.64)*** | -0.56  (-0.77, -0.36)*** | -2.83  (-4.92, -0.73)** | -6.00  (-9.52, -2.49)** |
| External environment |  |  |  |  |  |  |
| NDVI | 124.42  (64.14, 184.69)*** | -3.59  (-10.66, 3.47) | -2.22  (-15.30, 10.87) | -4.11  (-12.19, 3.97) | 241.94  (150.19, 333.68)*** | 376.44  (228.53, 524.35)*** |
| Nitrogen dioxide | -2.91  (-4.77, -1.06)** | 0.16  (-0.05, 0.38) | -0.04  (-0.43, 0.35) | 0.09  (-0.15, 0.34) | -2.89  (-5.54, -0.23)* | -5.64  (-10.06, -1.21)* |
| Atmospheric water vapour | -0.04  (-0.16, 0.07) | 0.02  (0.00, 0.03)* | 0.04  (0.02, 0.07)** | 0.01  (-0.01, 0.03) | -0.18  (-0.35, -0.02)* | -0.22  (-0.50, 0.06) |
| Formaldehyde | 9.37  (-72.12, 90.86) | -9.62  (-19.36, 0.12) | -1.40  (-19.18, 16.38) | -8.39  (-19.30, 2.52) | -18.79  (-136.13, 98.55) | -27.17  (-222.37, 168.04) |
| Ozone | 75.19  (18.35, 132.04)** | -0.94  (-7.60, 5.71) | 7.58  (-4.64, 19.81) | 8.25  (0.74, 15.77)* | 82.35  (0.43, 164.26)* | 168.32  (31.61, 305.03)* |
| PM2.5 | -3.16  (-6.57, 0.25) | 0.39  (-0.02, 0.79) | 0.35  (-0.38, 1.09) | 0.26  (-0.19, 0.71) | -2.70  (-7.59, 2.19) | -4.85  (-12.99, 3.29) |
| Maximum LST | 0.33  (-0.71, 1.37) | 0.00  (-0.12, 0.13) | 0.12  (-0.10, 0.35) | -0.05  (-0.19, 0.09) | -0.60  (-2.09, 0.90) | -0.43  (-2.92, 2.06) |
| Median LST | -1.45  (-3.46, 0.57) | 0.05  (-0.19, 0.29) | 0.72  (0.26, 1.18)** | 0.05  (-0.23, 0.32) | -3.56  (-6.49, -0.62)* | -4.96  (-9.81, -0.11)* |

**References:**

1.	Cope, M., et al., *Sydney particle study-stage-II*. 2014: CSIRO Marine and Atmospheric Research.

2.	Knibbs, L.D., et al., *A national satellite-based land-use regression model for air pollution exposure assessment in Australia.* Environ Res, 2014. **135**: p. 204–11.

3.	Veefkind, J.P., et al., *TROPOMI on the ESA Sentinel-5 Precursor: A GMES mission for global observations of the atmospheric composition for climate, air quality and ozone layer applications.* Remote Sensing of Environment, 2012. **120**: p. 70–83.
